# The decision-making processes of UK general practice nurse prescribers when managing acute illness in patients with multimorbidity and polypharmacy: a qualitative study using think aloud and staged vignettes

**DOI:** 10.1017/S1463423626100826

**Published:** 2026-02-02

**Authors:** Annie Herklots, Sue Latter, Chris McLean

**Affiliations:** University of Southampton – Highfield Campus: University of Southampton, Southampton, UK

**Keywords:** complexity, decision-making, general practice, multimorbidity, nurse prescribers, polypharmacy

## Abstract

**Aim:**

To investigate the decision-making processes of nurse prescribers in general practice when managing acute episodes of illness in patients with multimorbidity.

**Background:**

Nurse independent prescribers in UK general practice are facing increasing complex clinical decision-making when assessing patients presenting acutely with undifferentiated and undiagnosed conditions as multimorbidity and polypharmacy becomes increasingly common. This qualitative study investigated the decision-making processes of nurse prescribers in general practice when managing acute episodes of illness in patients with multimorbidity.

**Methods:**

Fourteen general practice nurse prescribers were recruited through purposive sampling. Think aloud in response to staged vignettes was used followed by semi-structured interviews. Thematic analysis was used to analyse think aloud and interview data.

**Findings:**

Participants were experienced nurses with a range of clinical exposure and training who mostly made appropriate diagnostic and prescribing decisions. Pockets of expertise were revealed which reflected participants’ clinical experience, but there was a high rate of referral to the GP for some vignettes. Participants’ decision-making was underpinned by both analytical and intuitive processes, the quality of which was dependent on their individual knowledge and experience. A reliance on pattern recognition, aligned to intuitive decision-making, to determine the content of the consultations was identified as an area of risk and showed all participants to be inconsistent in their identification of complex factors. Omission of these factors could have important implications for prescribing decision-making. Organizational issues such as time-limited clinics also shaped the content of participants’ consultations, encouraged a limited, problem-focused approach, and reduced the opportunity for mentorship. Comprehensive knowledge, clinical experience, and mentorship are critical to ensure nurse prescribers make optimal decisions in the context of patients with multimorbidity. A team approach to the management of acute presentations in these patients is recommended to improve patient experience and maximize nurse prescribers’ contribution to the general practice workforce.

## Background

General practice is facing significant challenges both in the UK and internationally with increased work load causing stress and burn out amongst younger doctors whilst increasing numbers of older, more experienced doctors plan to leave the work force (Gunja *et al.*, 2022). This is on the background of a global shortage of doctors (Lawson, 2023). Declining general practitioner (GP) numbers in the UK have necessitated a move towards a multidisciplinary general practice-based workforce in which the development of general practice nursing is recognized as a key contributor (Royal College of General Practitioners, 2022; NHS England, 2023).

Nurse independent prescribing in the UK has developed considerably over the years since its inception in 1992 and appropriately trained nurses can now prescribe any drug for any medical condition with the exception of some controlled drugs for the treatment of addiction (National Institute of Health and Care Excellence, 2025). UK general practice nurses have increasingly taken on roles traditionally undertaken by the medical profession such as diagnosis and prescribing and are facing increasingly complex clinical decision-making in a society where multi-morbidity, and consequently polypharmacy, are common in adults over the age of 65 (National Institute for Health Care and Excellence, 2015). Historically, general practice nurses have evolved into these roles from more traditional nursing roles typified by wound care, immunizations, and chronic disease management. Such roles have been characterized by NHS England in their framework for general practice nursing which differentiates between nurses working at enhanced and advanced levels (Health Education England, 2021). A defining feature of advanced level nurses in this document is being skilled in the assessment and management of patients presenting with undiagnosed and undifferentiated conditions and having a masters’ level qualification, whilst the descriptor of enhanced level nurses is more reflective of those who have specialist knowledge in chronic disease management. Importantly an overlap between the two roles is recognized where some nurses at the enhanced level may develop ‘advanced’ clinical aspects of their role, and this is representative of many nursing roles that have developed over time in general practice (Health Education England, 2021). The framework suggests several nursing roles appropriate to each level and aligns the role of ‘nurse practitioner’ to enhanced level nurses and ‘advanced nurse’ to advanced level nurses. However, both in the UK and internationally there is confusion regarding titles given to nursing roles and their associated scope of practice and training (Leary *et al.*, 2017). Additionally, the titles ‘nurse practitioner’ and ‘advanced nurse practitioner’ are unprotected by law in the UK, unlike other countries such as Australia and Canada where the title nurse practitioner is protected. Consequently, these titles are used to represent a variety of roles and associated scopes of practice in the UK (Leary *et al.*, 2017)

Although there are moves by the NMC to regulate advanced practice (Nursing and Midwifery Council, 2025a) and a number of capability frameworks published (Health Education England, 2021; NHS England, 2025b), to date there has been no mandated training for enhanced or advanced practice. Therefore the only common post-registration training requirement for nurse prescribers in enhanced and advanced roles is a prescribing qualification, and whilst appropriate assessment and diagnostic skills are a pre-requisite to enrolment on the prescribing programme, the required level of training in these skills is not specified (Nursing and Midwifery Council, 2024b). Consequently, these nurses who are usually considered expert in general practice nursing, may be novices when taking on the more advanced aspects of these roles, specifically the complex decision-making associated with diagnosis and prescribing (Brook and Rushforth, 2011).

Dual process theory, has been widely applied to clinical decision-making and recognizes that a combination of both analytical and intuitive processes is used to solve problems and make medical diagnoses (Croskerry *et al.*, 2017). Dual process theory identifies cognitive processes that are quick, automatic, and intuitive such as pattern recognition, and those which are slow and analytical (Evans, 2008). Croskerry (2009b) developed a model of diagnostic reasoning which exemplifies the interchange of intuitive (System 1) and analytical (System 2) processes in diagnostic reasoning and highlights the potential strengths and weakness of both systems and the risks associated with the use of intuitive processes. Intuitive processes rely heavily on the individual’s experience and utilize heuristics or mental short cuts making them efficient when applied by experts but prone to error, which may have catastrophic implications for patients (Croskerry, 2009c). Intuitive reasoning, with little reliance on analytical processes, is associated with expert practice in nursing (Benner, 1984) however, a reliance on intuitive processes informed by nursing experience to inform diagnostic and prescribing decisions in situations of complexity may be inappropriate.

Studies investigating nurse practitioners’ decision-making without a focus on multimorbidity and across a range of settings, found that both analytical and intuitive processes were used (Offredy, 1998; Offredy *et al.*, 2008) with some studies indicating that where intuitive processes were used by clinicians with insufficient experience there was a risk of inappropriate decision-making (Offredy *et al.*, 2008; Pirret *et al.*, 2015). Whilst early studies have shown nurse prescribers to be safe and effective prescribers for patients with a range of clinical conditions (Latter *et al.*, 2010; Naughton *et al.*, 2013; Weeks *et al.*, 2016), there is some suggestion from some studies without a focus on patients with multimorbidity, that prescribing for this group of patients is challenging for these nurses and may not always be appropriate (Naughton *et al.*, 2013; Maddox *et al.*, 2016; Williams *et al.*, 2017; Abuzour *et al.*, 2018). Despite this, there are limited studies investigating the decision-making processes of nurse prescribers, and to our knowledge none that focus on nurse prescribers in general practice managing acute presentations in patients with multimorbidity. Lack of research into this increasingly important area of nurse prescribers’ practice makes investigating their decision-making processes, of prime importance

## Aim

The aim of this study was to investigate the decision-making processes of nurse prescribers in general practice when managing acute episode of illness in patients with multimorbidity and explore how nurses justify and explain their decision-making.

## Method

This was a qualitative study using think aloud, vignettes, and semi-structured interviews. Vignettes, or written clinical scenarios, combined with think aloud, in which participants are asked to verbalize their thought processes, have been shown to be a useful tool to investigate clinical decision-making (Evans *et al.*, 2015; Thompson *et al.*, 2017; Abuzour *et al.*, 2018). An inductive process of thematic analysis informed by Braun and Clarke’s (2006) framework was undertaken to analyse both think-aloud and interview data.

## Recruitment of participants

Fourteen general practice nurse prescribers who regularly managed acute illness presentations in adults with multi-morbidities in general practice were recruited from GP practices in the south of England. Potential participants were contacted via email through the practice manager of over 180 individual GP practices in nine clinical commissioning groups. Recruitment continued until data saturation was achieved.

## Data collection

Three methods were used for data collection; think aloud in response to four vignettes and semi-structured interviews. A novel use of vignettes was piloted and used in this study. Vignettes were designed to capture the range of clinical information that might be sought by participants to support their decision-making during the course of the consultation. Review of the literature revealed that in other studies using think aloud and vignettes to investigate decision-making of nurse prescribers, the vignettes had very little requirement for participants to request information and participants were usually presented with a complete scenario at the outset (Pirret *et al.*, 2015; Abuzour *et al.*, 2018). The acquisition of information to support decision-making in patients with multimorbidity and polypharmacy is an important element of the consultation, and by supplying a complete vignette without a requirement for participants to request this information, decision-making processes could not be comprehensively explored, and there was a risk of this vital component being overlooked.

Four vignettes representing typical examples of acute presentations to general practice in patients with multimorbidity and polypharmacy were composed by AH, an experienced advanced nurse practitioner (ANP) in primary care (See Appendix 1 for summary of vignettes). The format of each vignette was based on a clinical consultation which was divided into stages to represent the acquisition of information within a consultation Information relevant to each stage was represented on separate cards e.g. physical examination, social history, observations. By staging the consultation in this way participants were required to determine the information they needed to undertake the assessment (see Appendix 2 for example vignette). The vignettes were reviewed by two experienced clinicians for face and content validity and the process of think aloud and interviews was piloted with two experienced advanced nurse practitioners. Minor amendments were made in response to their feedback.

Data collection took place between May 2019 and February 2020. The think aloud and semi-structured interviews were conducted at a place of the participants’ choice ensuring that there was access to resources such as drug formularies that would normally be available. Semi-structured interviews took place immediately after the think aloud stage and focused on reflections on decisions prompted by the vignettes as well as participants’ typical decision-making with patients presenting with acute presentations on a background of multi-morbidity and polypharmacy in practice (see Appendix 3 for interview schedule). Interviews lasted between one to two hours and were conducted by author, AH. Interviews were audio recorded, and all data were anonymized.

## Data analysis

Data analysis was informed by Braun and Clarke’s (2006) framework of thematic analysis which was applied to both think aloud and interview data. Interviews were transcribed verbatim. Line by line coding was undertaken (by AH) for the think aloud and interview data for each participant.

Coding of think aloud data included pictorial representation of the information collected and the judgements and decisions made by each participant for each vignette. This enabled comparison of the structure and content of consultations between and within participants’ consultations, and judgements to be made regarding the decision-making processes utilized. In addition, each participant’s response was analysed to see to what extent they addressed the key clinical issues embedded in each vignette and the outcomes of diagnostic and prescribing decision-making were compared to what was considered to be the optimal response for each vignette. These were determined through face and content validity review of the vignettes and during the pilot process.

Codes from analysis of think aloud were considered alongside those generated from the semi-structured interviews in order to confirm or develop initial codes. Emerging and evolving themes were discussed with authors SL and CM to develop sub-themes and four over-arching themes.

## Rigour and reflexivity

Rigour was maintained with reference to Lincoln and Guba’s (1985) evaluative criteria throughout to ensure the maintenance of trustworthiness. It was acknowledged that whilst author AH’s clinical background may provide valuable insights into the research area and facilitate interpretation of the participants’ experiences there was a risk of researcher bias. This occurs where the researcher’s pre-existing beliefs are not challenged, and there is a failure to look beyond their own experience (Dodgson, 2019). To ensure codes were generated from the data and driven by the language used by participants, and to protect against any preconceived ideas from the author’s background, memos were made during analysis of the data and a reflexive diary maintained throughout the process (Lincoln and Guba, 1985).

## Findings

### Participants

Fourteen nurse prescribers were recruited. All were female and had considerable nursing experience with the majority qualified for over twenty years. Most participants described themselves as ‘nurse practitioners’ whilst others used the term ‘advanced nurse practitioner’; this did not necessarily correspond to associated qualifications or the scope of the role. Details of clinical experience and qualifications are shown in Table 1. There was no single common trajectory to the advanced nurse practitioner/nurse practitioner role. For some the transition had evolved through working as a practice nurse and for others it was a new primary care role, for which they drew on previous experience from other settings. There was no uniformity to their qualifications, but most had undertaken some additional training to support their role.

**Table 1.** Summary of participant characteristics

		Participants (all female)
Age range	35–44	1
	45–54	7
	55–65	6
Number of years as a nurse prescriber	<3 years	2 (one of these participants also had <3 years’ experience in the nurse practitioner role)
	3–10 years	6
	>10 years	6
Number of years in advanced nurse practitioner/nurse practitioner role	<3 years	5
	3–10 years	5
	>10 years	4
Total years primary care experience	<3 years	0
	3–10years	4
	>10 years	10 (three of these participants also had >10 years’ experience in ANP/NP role and as NIPs)
Post registration qualifications	MSc	3
	Post registration degree	4
	Individual modules*	6
	Post registration diplomas	6 (in addition to modules/post reg degree)
	No relevant post registration qualifications	1

* Includes university modules: history taking and physical assessment, diagnostic decision making, research methods, minor illness, transition to advanced practice.

## Clinical appropriateness of responses to the vignettes

Most participants reached the anticipated diagnosis of each vignette independently, however there was considerable variation in the differential diagnoses generated in the diagnostic process and in participants’ consideration of diagnoses indicating serious disease or ‘must not miss’ diagnoses.

Overall prescribing decisions were clinically appropriate, but there was a high rate of referral to the GP for some vignettes (Table 2). Some participants chose alternatives to first line choices recommended by prescribing guidelines but mostly these prescribing decisions were justifiable. A minority of decisions would have presented a potential risk to the patient; for example, the decision by one participant to prescribe ibuprofen for a patient on warfarin.

**Table 2.** Participant independence in prescribing decision-making

Total number of participants (P) who undertook vignettes*	Vignette 1 (13 participants)	Vignette 2 (14 participants)	Vignette 3 (14 participants)	Vignette 4 (14 participants)
Completed independently	12 (P3,4,5,6,7,8,9,10,11,12,14)	9 (P2,3,4,6,7,8,11,12,14)	2 (P3,14)	10 (P3,4,6,7,8,10,11,12,13,14)
Completed independently but made plan in conjunction with specialist team or secondary care	0	0	3 (P1,7,8)	0
Refer/liaise with GP for management	3 (P2,13 advice re hypertension)	5 (P1,5,9,10,13)	9 (P2,4,5,6,9,10, 11,12,13)	4 (P 1,2,5,9)

* Data from Participant 1 in response to Vignette 1 was used as pilot data and therefore was not included in the main study.

Issues of complexity such as medication adherence and management of co-morbidities were inconsistently addressed by participants (Table 3) and consequently the impact of these on prescribing decision-making was overlooked.

**Table 3.** Complex factors identified by participants

	Social situation	Management co-morbidities	Medication adherence
Vignette 1	9 Participants (P2,4,6,7,9,10,12,13,14)	5 Participants (P2,6,10,13,14)	6 Participants (P2,6,10,12,13,14)
Vignette 2	3 Participants (P1,2,14)		2 Participants (P1,2)
Vignette 3	3 Participants (P2,7,13)		3 Participants (P2,7,13)
Vignette 4	10 Participants (P1,2,4,5,7,9,10,12,13,14)	6 Participants (P1,3,6,7,814)	1 Participant (P1)

* Data from Participant 1 in response to Vignette 1 was used as pilot data and therefore was not included in the main study.

Four overarching themes were established through analysis of think aloud and interview data. These were as follows: scope and content of consultations, diagnostic decision-making processes, prescribing decision-making processes, and influences on decision-making.

### Scope and content of consultations

All participants were found to follow a basic structure of history, examination, diagnosis, and management/treatment, but the scope and content of the consultation varied between the participants with some identifying and collecting more cues and making more judgements and decisions than others. The consultation was not a linear process with participants revisiting the history and examination stages frequently. This approach suggests that the participants used the information they were gathering to determine the scope and content of the consultations rather than using a formalized structure or check list. The fluidity of this process is described by Participant 9 below.

*‘I take the history in quite a chatty way usually and we flit backwards and forwards. I’m not particularly structured but I’m aware that I follow a structure to it, so I am taking their past, present and other complaints, but I flit backwards and forwards between it and then I do tend to always come back and clarify with them, and I will revisit some areas several times during a consultation.’ (Participant 9, interview)*

All participants focused on the presenting complaint, but there was variation in their collection of cues that identified complex factors such as adherence to medication, management of co-morbidities, and medication review. These aspects of the consultation were addressed inconsistently amongst the participants with the majority overlooking some of these issues and non-adherence to medication being most frequently omitted.

Most participants relied on intuitive processes in which pattern recognition generated from cues such as observations of vital signs were relied on to prompt further exploration. Even then, this did not always prompt enquiry beyond the context of the presenting complaint. This is exemplified in the different approaches taken by two participants in response to the patient’s raised blood pressure in vignette one. Participant 5 is shown to interpret this finding in the context of the patient’s presenting complaint of shingles, whilst Participant 14 was prompted to consider wider issues such as non-adherence to their medication.

*‘His obs (observations) are fine. Blood pressure is up a bit. His blood glucose is a bit up and his SATS are down a bit. How old is he? 74. So that to me wouldn’t be ringing too many alarm bells. (Participant 5, vignette 1, think aloud)*

*‘Yes, so he’s apyrexial pulse is fine, his blood pressure is raised so then I would ask him if he’s been taking all the medication he’s been prescribed and if he wasn’t taking the amlodipine if there was a reason for it.’ (Participant 14, vignette 1, think aloud)*

This reliance on intuitive processes to identify issues representing complexity rather than taking a more structured analytical approach, risks overlooking important factors within the consultation that may influence prescribing decision-making and could also raise issues of patient safety. For example, in the above example Participant 5 not only omitted to consider the management of the patient’s hypertension, but by failing to identify that the patient was non-adherent to their medication did not consider the implications for this when prescribing for the presenting condition which required medication to be taken five times a day.

### Diagnostic decision-making

Diagnostic decision-making was underpinned by pattern recognition, an intuitive process which informed hypothesis generation. Participants recognized familiar patterns from clustering cues from the history and examination which prompted them to make an intuitive judgement regarding the patient’s presentation.

*‘And he’d say OK I’ve got a pain in the right side of my chest; I’ve had it for a week, and I’ve got spots on the right side of his chest over the last few days. Painful. Do you want my thinking straight away? Shingles.’ (Participant 2, vignette 1, think aloud)*

Participants identified pattern recognition as a process they used in their diagnostic decision-making

*‘I suppose when you are looking to make diagnosis you are looking at patterns. So, you are looking for historical patterns and you are looking for clinical patterns. And you put those together.’ (Participant 8, interview)*

However, participants used both intuitive (pattern recognition) and analytical (hypothesis deduction) in their diagnostic decision-making. Participants were aware of the potential pitfall of relying on their first intuitive hypothesis and failing to think widely enough about differential diagnoses and were shown to use metacognition, a process of actively reviewing their decision-making processes, to ensure they remained open-minded and avoided drawing conclusions too early.

*‘I’m immediately thinking shingles at this moment.*

*Yes, but I wouldn’t say it out loud and I’d keep it inside and I’d keep my mind open because so many times you jump to a conclusion and then it’s really not that.’ (Participant 3, vignette 1, think aloud)*

Participants continued with an analytical process of cue gathering to test their hypothesis which prompted the generation of further hypotheses. Pattern recognition therefore initially contributed to an analytical process of differential diagnosis in which further hypothesis testing was undertaken. Participants took an analytical approach and looked for cues that either increased or decreased the likelihood of a diagnosis until they arrived at a working diagnosis.

*‘Using three pillows at night, inhaler not helping much although they don’t often with the COPD do they? Sputum not changed so you are thinking not chest infection. Chest not sounding wheezy by the sound of it. And the fact that he’s swelling up that would make you think maybe he’s just filling up with fluid really.’ (Participant 12, vignette 3, think aloud)*

Participants did not access any resources to support their diagnostic decision-making and relied on their own knowledge and experience to generate hypotheses. This was reflected in the variation in the range of differential diagnoses generated by participants.

There was inconsistency in participants’ consideration of ‘must not miss’ differentials within the consultations. This was clearly shown in responses to two of the vignettes in which less than half the participants considered potential serious illness differentials in either vignette, and only one participant considered these in both. Those participants who considered serious illnesses appeared to do so either through intuitive responses to specific cues in the consultation or as part of an analytical process of ruling out serious disease.

### Prescribing decision-making

The majority of prescribing decisions comprised both analytical and intuitive processes. Participants were seen to weigh up the need for treatment and then most relied on recall to identify the appropriate drug. Recall represented an intuitive process in which participants rapidly recalled the appropriate treatment for the condition. This process is exemplified in the example below in which Participant 3 considered the treatment for shingles.

*‘So, yes, I’d be thinking shingles. Then I’d be thinking, you are wanting my prescribing thoughts, don’t you? I’d be thinking about Acyclovir 800mgs, five times a day for five days.’ (Participant 3, Vignette 1, think aloud)*

Although participants relied on intuitive processes to recall the appropriate drug, this was in the context of an analytical process in which they reviewed their decision-making to consider allergies and drug interactions and adapted their choice.

*‘I would treat her with some antibiotics, so, depending on what they clash with, if she’s not allergic, oh she’s allergic to Penicillin so I would probably go for Doxycycline.’ (Participant 6, vignette 4, think aloud)*

The majority relied on the electronic prescribing system to alert them to potential drug interactions.

*‘So I’d give him his prescription for that but bearing in mind what else he’s on I would pray to the gods that the computer will flag up to me if there were any interactions.’ (Participant 10, vignette 1, think aloud)*

and they then drew on their knowledge and experience, and at times referred to resources such as drug formularies or evidence-based guidelines (Braun and Clarke, 2006), to inform their decision-making.

Analytical processes were also used by participants to determine whether they felt competent to prescribe for a patient and participants were seen to weigh up the complexity of the prescribing scenario and determine if they would continue to undertake the decision-making independently.

*‘I mean sometimes you are at a loss as to what to prescribe because actually everything looks like it interacts with something and there’s no easy answers there and with people with heart failure it’s quite a difficult process if their kidney function is a bit knocked off. You are trying to improve the heart failure but then you might knock off the kidneys and those things do get very complex in which case you should seek some help from the GP or a specialist team.’ (Participant 2, vignette 3, think aloud)*

Mostly participants made appropriate prescribing decisions and identified areas where they did not have sufficient knowledge or experience to independently make the prescribing decision. The electronic prescribing system acted as an additional support to analytical processes and prompted participants to consider interactions and drug allergies. However, there were a few instances where suboptimal prescribing decisions were made in both intuitive (recall – see example of shingles treatment above with suboptimal duration) and analytical processes which could be attributed to insufficient underpinning knowledge and lack of experience in prescribing a particular drug.

### Influences on decision-making

The major influence informing participants’ decision-making was reported to be their individual scope of practice. Several factors were identified by participants as contributory and influential to this which could be broadly categorized under participant and organizational factors.

#### Participant factors

Key for participants was their confidence and perceived competence to manage the scenarios which was dependent on their previous clinical experience, exposure to similar prescribing scenarios and their underpinning knowledge.

*‘It is a big responsibility and that’s why you need to work within your level of confidence and competence really and accept when you need to go and ask for help’ (Participant 1, interview.)*

Participants considered prescribing decision-making to be risky where the potential for harm to the patient was perceived as unacceptably high or the decision was one for which they felt they had insufficient knowledge, and the consequences of prescribing may have repercussions for them with their professional regulatory body (in the UK, the Nursing and Midwifery Council).

*‘What stops me [prescribing]? My PIN (professional identification number) and wanting to be safe.’ (Participant 4, interview)*

There was variation amongst the participants with regard to which vignettes and to what extent they were prepared to independently manage them although some vignettes proved more challenging than others to the majority of participants. This extended beyond the cumulative level of experience in the role with some very experienced prescribers completing some but not all vignettes independently, and similarly, some relatively inexperienced prescribers successfully completing some vignettes independently. This was explained not by the length of their experience, but the type of experience they had had in previous roles and specialties, and how this related to the presenting conditions within the vignettes. This was exemplified in Vignette 3 which focused on a patient with decompensated heart failure and was a condition for which the majority of participants did not feel they had sufficient experience to make prescribing decisions.

*‘So, I’m not at the point where I would start changing heart failure med and increasing diuretics. No, I recognise that that’s not where I’m confident. There are other ANPs who have got a background in heart failure. I have a colleague who was a heart failure specialist so she would feel quite happy to you know, to stop this and change that, but no, then I think, it’s for me, you think OK I’ll speak to the team.’ (Participant 1, interview)*

Participants identified the importance of drawing on clinical experience in previous roles in their ability to manage the vignettes. This was important in determining the clinical conditions that they felt confident to tackle.

*‘I can do the insulins and all that sort of stuff because when I was a practice nurse years ago, I specialised in diabetes and my background is in cardiac ICU, so I’m happy with all those.’ (Participant 3, interview)*

Where participants did not feel confident and competent, they referred the patient to the GP and were unwilling to undertake prescribing decisions where they considered there to be an unacceptable risk of potential harm to the patient and risk their professional registration.

*‘I think in an 86-year-old I wouldn’t be looking at something like colchicine because it’s a very high toxic drug and I definitely wouldn’t prescribe that without talking to a GP.’ (Participant 14, vignette 2, think aloud)*

#### Organizational factors

The majority of participants reported that they ran clinics with appointments that were booked on the day with 10-15 minutes allocated for the consultation. Participants commented on the impact of time-limited appointments in determining the content of their consultations and identified that more time was needed to fully assess complex patients. Complex patients were consistently identified as disrupting the flow of the clinic and required more time and were a consistent source of stress amongst participants.

Some participants described how time would also influence the depth of their consultation. Time restrictions reportedly led to a problem-focused approach in which participants prioritized the presenting complaint and relied on intuitive processes to identify additional complex factors within the consultation. For example, the participant below admits that they would only ask about social circumstances if they had enough time left in the appointment.

*‘Depending on the time, how much longer I’ve got in the consultation, I would ask him what support he’s got at home and if that’s the case I would arrange that during the next review.’ (Participant 9, vignette 1)*

Development of clinical knowledge was reported to be self-directed but influenced by the environment in which participants worked. Time restrictions were reported to impact learning and mentorship opportunities which for most participants was opportunistic and dependent on available time. A tension was identified between utilizing a learning opportunity and managing time, and this influenced participants’ decisions whether to ask the GP for advice or refer the patient to their list.

*‘So, there are two ways depending on the pressure of our clinic and the fact that I am forever, this is still a learning process for me, if they (the GPs) can they actually come into the room so that I can overhear what’s actually happening next. But occasionally I, well actually just depending on pressures of the day, they may need to go back into the waiting room knowing that actually they’re next on the list.’ (Participant 13, interview)*

Support from GPs was mixed with some participants describing a supportive relationship whilst others worked more independently and had little contact with GPs. A few participants described a team-based approach to patients with support and mentorship more readily available to them.

## Discussion

This study has identified that overall nurse prescribers used a combination of intuitive and analytical processes and made appropriate diagnostic and prescribing decisions. Pockets of expertise were identified which reflected the individual participant’s clinical background and experience. However, consultations were often not comprehensive and a reliance on intuitive processes exemplified by pattern recognition to identify complex factors, represented an area of risk. Where participants considered themselves to have insufficient knowledge or experience to complete a consultation they referred to the GP. Organizational factors further influenced decision-making where for most participants time-limited appointments encouraged a problem focused approach and limited opportunities for mentorship.

Diagnostic and prescribing decision-making were mostly appropriate and were characterized by the use of both intuitive and analytical processes. Participants drew on their knowledge and experience to inform these processes which was enabling in the management of some vignettes, but the diversity of participants’ clinical experience and training meant that there was considerable variability in their ability to independently complete individual vignettes.

Pattern recognition is commonly used by nurse practitioners and GPs in diagnostic decision-making (Thompson *et al.*, 2017; Abuzour *et al.*, 2018) and is strongly dependent on the experience of and exposure to a particular condition over time. Where this is insufficient there is the potential for error (Croskerry, 2009a). In our study, participants’ reliance on pattern recognition to generate hypotheses as part of the process of differential diagnosis meant that there was a wide range of differentials considered by participants. Although mostly the final diagnosis was appropriate, the lack of consistent approach to ruling out ‘must not miss’ diagnoses was concerning. ‘Must not miss’ diagnoses were not considered by over half the participants in two of the vignettes. Actively ruling out serious illness and searching for red flags within the consultation is of key importance in respect of patient safety (Silverston, 2020). Consultation models such as the Calgary-Cambridge model (Kurtz *et al.*, 2003) are widely taught and utilized in advanced practice (Diamond-Fox, 2021) A more considered, structured approach to this aspect of the consultation using a consultation model could be indicated.

Participants also relied on pattern recognition to identify complex factors within the vignettes, however this was shown to be unreliable, and important factors such as medication adherence were frequently overlooked by participants. This suggests that this mode of thinking may not be appropriate for the range and complexity of patients presenting to general practice and that nurse prescribers may lack sufficient experience to undertake this approach for all presentations. Time limited appointments both encouraged this mode of thinking and contributed to participants adopting a problem focused approach. This behaviour is also reflected in studies of GPs’ management of patients with multimorbidity where a satisficing approach was often adopted in which only acute problems were tackled due to time restrictions (Damarell *et al.*, 2020). This suggests that longer appointment times for patients with multimorbidity would benefit all of the primary care team.

Participants referred to GPs where they did not consider they had the confidence and competence to manage a presentation exemplified by the vignette. Whilst working within boundaries of competence is key to patient safety, in practice this onward referral of complex patients has implications for increasing demand on GPs. Central to the policy drive to diversify the primary care workforce is a decline in the number of GPs and an assumption that diversification will reduce GP workload (Royal College of General Practitioners, 2022). However, recent research into pharmacist–GP collaboration and skill mix change in primary care has also shown that employment of the former does not necessarily reduce the workload for the latter (McDermott *et al.*, 2022; Park, 2024). Therefore, our findings show that caution in making this assumption is required at this time.

## Implications for practice

Matching clinician skill to the patient’s problem has been shown to be of key importance in creating additional capacity in general practice (McDermott *et al.*, 2022). Pockets of expertise based on previous clinical experience were identified amongst the participants in our study. Directing this expertise to appropriate patients could improve efficiency and patient safety. The working practices of most study participants determined that they worked independently from an individual patient list, but this was not always efficient, resulting in referral to the GP for some complex situations. With declining GP numbers and rising patient demand, findings from this study indicate that this traditional way of working might be reviewed. A minority of participants in this study described a team-based approach to the patient list which enabled flexibility in consultation times, appropriate allocation of skills, and encouraged mentorship and support from GPs. For complex patients, a team-based approach might also enable greater continuity of care with a practitioner known to them, which is shown to improve clinical outcomes and reduce consultation time (Damarell *et al.*, 2020). However, this is clearly challenging to achieve with the current workforce crisis and in response to this some UK general practices have adopted micro-teams, an approach common in US models of care, that allocate several members of the multi-disciplinary team led by a named GP to a patient group (Baird *et al.*, Baird, 2020; Coombs *et al.*, 2023). This approach is in line with the NHS Long Term Workforce Plan (2023) (NHS England, 2023) which recognizes the need to support and develop multidisciplinary teams to manage complexity. Such teams may help to overcome both the discontinuity in patient care and the potential time burden for patients if nurses are unable to deal with their presenting condition and need to refer on to a GP. Micro-teams may be operationalized in general practices by connecting the patient with the team member with the most appropriate skills and ensuring that if the patient reconsults that the team member who knows them best and has the most appropriate skills is allocated (General Medical Council, 2025).

Time-limited appointments identified by study participants reduced the opportunity for them to seek support and develop their practice. Yet, the importance of clinical support from colleagues in developing prescribing practice is well documented in the literature (McIntosh *et al.*, 2016; Djerbib, 2018; Evans *et al.*, 2020). Development of expertise is only achieved if there is the opportunity to develop knowledge through appropriate feedback (Croskerry *et al.*, 2017) and therefore support from GPs, particularly in complex decision-making, is fundamental to equip primary care nurse prescribers to manage the array of presentations they encounter. A team approach using micro-team involving GPs, nurse prescribers and other members of the multi-disciplinary team could enhance the opportunity for nurse prescribers to develop their knowledge and scope of practice and share their expertise with other clinicians whilst improving patient outcomes.

## Implications for education

Internationally, advanced practice roles and associated training lack standardization and regulation (Wheeler *et al.*, 2022). Whilst NHS England have developed a multiprofessional framework for advanced practice (NHS England, 2025b) and accreditation of advanced practice programmes delivered by higher education institutes, these are not mandatory requirements, and advanced practice in the UK is currently not regulated. Whilst the NMC is in the process of considering steps to regulate advanced practice, including education and training (Nursing and Midwifery Council, 2024a) this is complex with standards not due to be published until 2027 (Nursing and Midwifery Council, 2025b). Furthermore, not all nurse prescribers in general practice are working as advanced practitioners. Mapped against the Health Education England, 2021 career and capability framework for general practice nurses (HEE, 2021) most participants in this study fall between enhanced and advanced level practice with their roles having a high clinical focus and, as such, would identify as nurse practitioners rather that advanced nurse practitioners and therefore would not be subject to regulation. Although the framework details core capabilities for different levels of practice, there is no jurisdiction in the Health Education England (2021) document regarding the appropriate continuing professional development (CPD) modules required by enhanced level nurses other than the specification that they should be aligned to the individual’s scope of practice.

Whilst pockets of expertise were identified in study participants, equally there were omissions and oversights in relation to the scope and content of the consultation. Preparation for nurse prescribers in general practice differs significantly from GP registrars who enter the role as qualified and experienced prescribers and are required to undertake an additional three-year period which is overseen by a GP trainer and includes rotation to hospital specialties and a rigorous system of examination (NHS England, 2025a). This may better equip GPs to manage multimorbidity and polypharmacy, although a systematic review (Damarell *et al.*, 2020) indicates that they too experience clinical uncertainty and face decisional risk in the context of complexity and multimorbidity. Nevertheless, our findings suggest that more consistent exposure to a more comprehensive range of clinical conditions may be a necessary pre-requisite for nurse prescribers in General Practice undertaking the assessment and management of acute presentations in patients with multimorbidity and polypharmacy. These recommendations should be tailored to global contexts beyond the UK where, despite the fact that internationally the education and legal status of nurse prescribing differs (International Council of Nurses, 2021), nurse prescribers, regardless of their individual prescribing authority and training, will encounter increasing complexity in an ageing population with associated polypharmacy and co-morbidities(World Health Organization, 2025).

The reliance in this study on intuitive processes to identify complexity indicates that a more structured and comprehensive approach to history taking which routinely encompasses issues such as medication adherence and social history would be beneficial in managing complex presentations. History taking is an essential skill of prescribers (Royal Pharmaceutical Society, 2021) and using a logical, structured approach is key to successful diagnosis and management (Butler, 2023). Furthermore, the risks associated with the use of intuitive processes identified in this study indicate that self-awareness and the ability to critical reflect on decision-making processes are highly valuable in addition to clinical experience, exposure, and mentorship when managing complex presentations. Although non-medical prescribing competencies demand a high level of diagnostic and prescribing decision-making there is currently no requirement to undertake a dedicated diagnostic or clinical reasoning module (Royal Pharmaceutical Society, 2021). NMC entry requirements for the prescribing programme stipulate that HEIs should confirm that applicants are at a proficient level in clinical assessment and diagnostic management (Nursing and Midwifery Council, 2024b) and whilst some Higher Education Institutions (HEI) demand completion of such modules prior to being accepted on to the programme, this is not universal. It therefore seems vital that where this is not an entry requirement, HEIs incorporate clinical reasoning into their NMP programmes.

Formalized and standardized training in assessment and diagnostic decision-making, alongside sufficient time to access mentorship and support is essential in order to ensure that nurse prescribers are sufficiently prepared to undertake comprehensive management of acute patients with multimorbidity and polypharmacy.

## Strengths and limitations

As far as we are aware this is the first study to evaluate nurse practitioners’ decision-making when faced with acute presentations in complex multimorbid patients presenting to primary care. This study has shown the value of vignettes and think aloud methods in the study of the decision-making processes. A unique and novel aspect of this study was the use of staged vignettes that allowed the sequential process of participants’ decision-making to be shown and evaluated, and employing think aloud revealed the processes that were used to determine what information was collected. Of significance was the contribution of staged vignettes to the important findings of this research which showed that some participants focused their data collection on the presenting complaint and the revelation that complex aspects of the patients’ histories were not always explored, revealing a dependence on intuitive processes to identify complexity within the vignettes by some.

As the participant sample was self-selecting it is possible that only those who felt sufficiently confident in their management of this group of patients volunteered to participate and therefore the findings may not be fully representative of all nurse prescribers undertaking this role. Future studies focusing on less experienced general practice nurse prescribers are recommended to address this issue.

## Conclusion

Whilst appropriate diagnostic and prescribing decisions focused on the presenting complaint were made by participants in this study, findings identified variations in nurse prescribers’ abilities to fully manage a range of complex patient scenarios. Pockets of expertise reflecting individual’s knowledge, experience and exposure to similar scenarios were revealed; in other instances, participants referred to the GPs for prescribing decisions. Whilst both intuitive and analytical processes were found to inform diagnostic and prescribing decision-making, a reliance on pattern recognition to determine complex factors within the consultation and the adoption of a problem-focused approach were identified as areas of possible risk.

A team approach to the management of acute presentations in patients with multimorbidity presenting to general practice is recommended alongside ready access to mentoring for nurse prescribers to ensure that their contribution to the general practice workforce is maximized and patient experience is enhanced.

## Data Availability

The qualitative data that support the findings of this study are not openly available. Participants did not consent to have their data shared.

## Appendix 1

### Summary of vignette presentations

(NB social history, physical examination and investigations are not included in this summary but were available on cue cards if requested by participants)

**Table:**

	Demographic details	Presenting complaint	Anticipated diagnosis	Co-morbidities	Medication	Drug allergies
**Vignette 1**	76-year-old male	Rash	shingles	Hypertension Type 2 Diabetes Asthma	Amlodipine Metformin Glipizide Salbutamol Clenil	No known drug allergies
**Vignette 2**	86-year-old male	Foot swelling	Gout	Hypertension COPD Aortic Valve replacement (2010) Mild dementia Depression Gastroesophageal reflux disease	Warfarin Mirtazapine Atorvastatin Bendroflumethiazide Omeprazole Salbutamol inhaler Spiriva Over the counter paracetamol and ibuprofen	No known drug allergies
**Vignette 3**	83-year-old male	Shortness of breath	Decompensated heart failure	Hypertension Myocardial Infarction Atrial fibrillation CKD 3 COPD Heart failure LVSD: Moderate/severe	Bisoprolol Ramipril Apixaban Atorvastatin Amlodipine BumetanideSalbutamolSeretide Spironolactone	No known drug allergies
**Vignette 4**	77-year-old female	Cough	Community acquired pneumonia	Type 2 diabetes Hypertension Ca breast (2006) Recurrent DVT Polymyalgia rheumatica	Ramipril Atorvastatin Metformin Warfarin Prednisolone Omeprazole	Penicillin

## Appendix 2

### Vignette 3

**Table:**

**CARD 1** **Initial information** 83-year-old male Shortness of breath
**CARD 2** **Computer summary** **PMH** Hypertension MI Atrial fibrillation CKD 3 COPD: Moderate Heart failure: LVSD: Moderate/severe **Medication:** Bisoprolol 2.5mg OD Ramipril 1.25 mg OD Apixaban 5mg BD Atorvastatin 80mg OD Amlodipine 5mg OD Bumetanide 2mg OD Salbutamol inhaler 2 puffs PRN Seretide 500 BD Spironolactone 25mg OD **Allergies:** None known **Ex-smoker**
**CARD 3** **General survey** With wife Alert, breathless walking into the room but settles at rest. Well perfused. Talks in full sentences
**CARD 4** **History of current complaint** Has been feeling more breathless over the week, especially on exertion. Has worsened Had a cold a couple of weeks ago and has a bit of a cough Afebrile Has been feeling more tired. Has tried using inhaler not helped much
**CARD 3** **Additional history** No colour change or increase of sputum Worse at night when lies down Using 3 pillows (usually 2) Inhaler hasn’t helped much No chest pains Ankles are more swollen
**CARD 4** **Adherence** Manages own medication Says he takes all medication as prescribed but mentions doesn’t take bumetanide when going out and only takes one normally
**CARD 5** **Social history** Lives with wife who is well Active member of local church Mobile with stick Alcohol 1–2 units daily
**CARD 6** **Vital signs** T 36.2 P 98 irreg RR 26 Sats 93% BP 100/50
**CARD 7** **Baseline observations** P 88 irreg Sats 95% BP 105/80 RR 24
**Card 8** **Physical examination** Chest: equal expansion, resonant, fine crackles to both lung bases Pitting oedema to mid-calf, right and left
**Card 9** **OTC meds** Nil
**Card 10** **Additional examination** Weight 80 kg (+3 kg) JVP + 4 cm
**Card 11** **Blood results** Last done 3/12 ago Stable FBC within normal range eGFR 41 Creatinine 129 Na 133
**Card 12** **Clinic letter from Heart Failure Team** Seen 3 months ago Diagnoses: MI 1993 Moderate/Severe LVSD Atrial fibrillation Current meds: Bisoprolol 2.5mg OD Ramipril 1.25 mg OD Apixaban 5mg BD Atorvastatin 80mg OD Amlodipine 5mg OD Bumetanide 1mg OD Salbutamol inhaler 2 puffs PRN Seretide 500 BD Spironolactone 25mg OD I reviewed this gentleman in clinic today. He reports feeling well and no specific increase in shortness of breath, no paroxysmal nocturnal dyspnoea and no chest pain. He does report some weight gain over the last few months. His ECG today showed AF with good rate control at 76 bpm. Echocardiogram showed impaired left ventricular systolic function with an ejection fraction of less than 40% Auscultation of his chest revealed fine bi-basal crackles. Pitting oedema was present to both ankles. JVP was not raised. Weight 78 kg This gentleman is generally stable although he may be mildly overloaded at present and could benefit from an increase in his bumetanide from 1–2 mg. He can be referred back to your routine care in the community.

## Appendix 3

### Interview schedule

**1. Ask participant for additional clarification from think aloud**

Prompts:

Which aspects did you feel confident in? Were there any particular issues within the vignettes that you found difficult?

Can you elaborate on how you reached these decisions?

On reflection was there any aspect you feel you could have explored further?

**2. Having completed the vignettes can you tell me a bit more about your decision-making when prescribing for acute presentations for patients with comorbidities and polypharmacy?**

Prompts:

How do you normally make such decisions? What are the processes involved?

How difficult do you find these decisions? If difficult, why is that?

Are there particular situations/presentations conditions that you feel more comfortable managing?

Why is this?

Can you identify how you have developed these skills? Training, peer support (GP, NIP), environment, prescribing culture

Are there aspects you find particularly challenging? If so, what are these?

What resources do you use?

Do you have any rules of thumb/short cuts you can identify?

How confident do you feel in managing these consultations independently?

**4. How often do you manage these kind of presentations independently?**

Prompts:

Seek advice, refer on, avoid?

**5. What resources do you find most useful in managing these kind of presentations?**

Prompts:

On-line resources, colleagues eg GP/pharmacists

**6. How well do you feel your prescribing training has equipped you to manage this group of patients?**

Prompts:

What particular aspects of training? Pharmacology lectures, DMP supervision?

What additional training support have you received?

What additional support would you find, or have you found beneficial?

**7. Can you tell me about your qualifications and clinical experience? Please do not identify organizations by name.**

Prompts:

Educational qualifications, clinical experience (organizations, length of time)
